# Supplementary material for: The causal role of circulating inflammatory markers in osteoporosis: a bidirectional Mendelian randomized study
Source: Front Immunol. 2024 Jul 18;15:1412298. doi: 10.3389/fimmu.2024.1412298 (PMC11291241; doi:10.3389/fimmu.2024.1412298)
Supplement: Supplementary file 1 [file DataSheet_1.zip › supplementary material/OSTEOPOROSIS-CIM/GCST90274759.tsv.gz_forest.pdf]

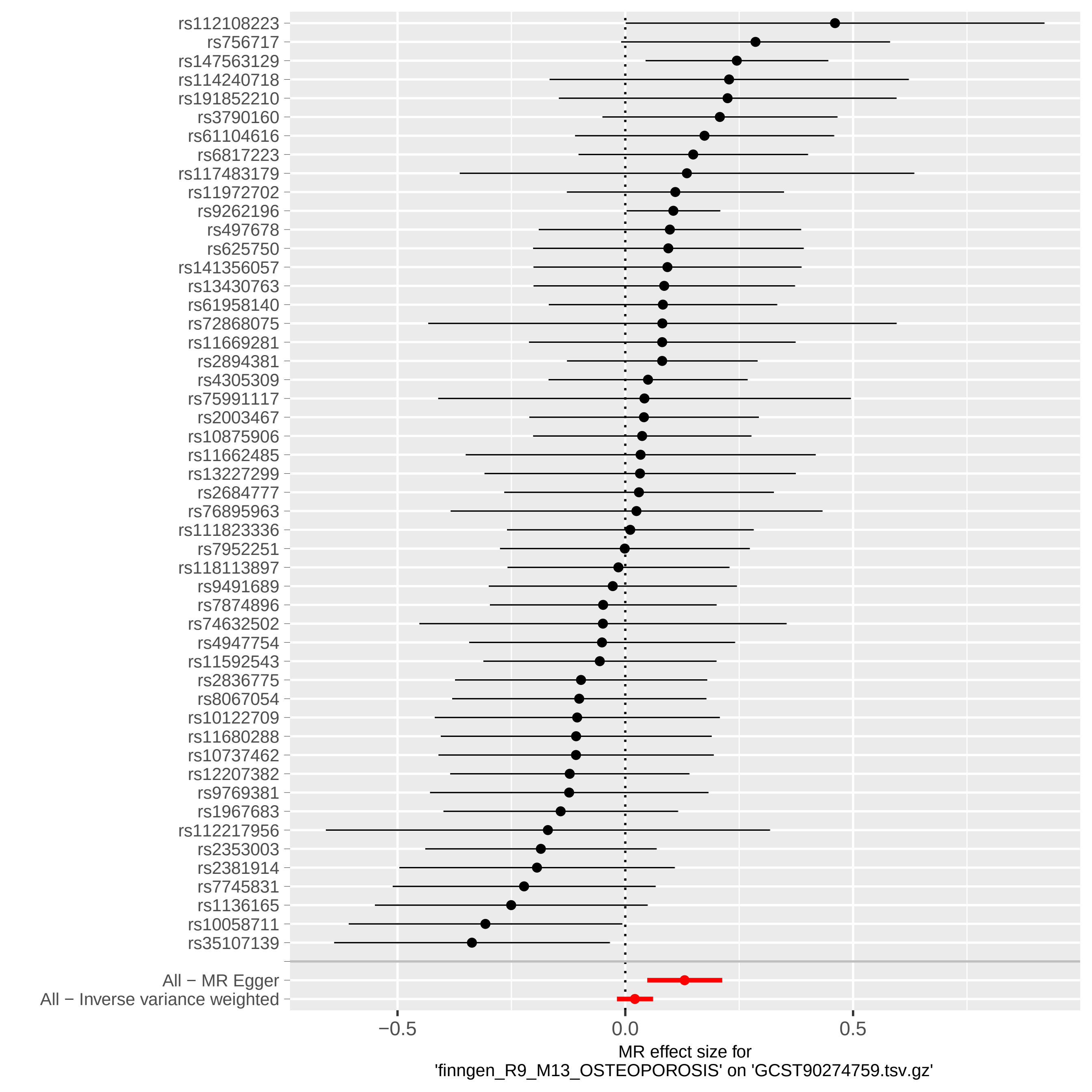
rs112108223
rs756717
rs147563129
rs114240718
rs191852210
rs3790160
rs61104616
rs6817223
rs117483179
rs11972702
rs9262196
rs497678
rs625750
rs141356057
rs13430763
rs61958140
rs72868075
rs11669281
rs2894381
rs4305309
rs75991117
rs2003467
rs10875906
rs11662485
rs13227299
rs2684777
rs76895963
rs111823336
rs7952251
rs118113897
rs9491689
rs7874896
rs74632502
rs4947754
rs11592543
rs2836775
rs8067054
rs10122709
rs11680288
rs10737462
rs12207382
rs9769381
rs1967683
rs112217956
rs2353003
rs2381914
rs7745831
rs1136165
rs10058711
rs35107139
All − MR Egger
All − Inverse variance weighted
−0.5
0.0
0.5
MR effect size for
'finngen_R9_M13_OSTEOPOROSIS' on 'GCST90274759.tsv.gz'
